# Geno2pheno_[HCV]_ – A Web-based Interpretation System to Support Hepatitis C Treatment Decisions in the Era of Direct-Acting Antiviral Agents

**DOI:** 10.1371/journal.pone.0155869

**Published:** 2016-05-19

**Authors:** Prabhav Kalaghatgi, Anna Maria Sikorski, Elena Knops, Daniel Rupp, Saleta Sierra, Eva Heger, Maria Neumann-Fraune, Bastian Beggel, Andreas Walker, Jörg Timm, Hauke Walter, Martin Obermeier, Rolf Kaiser, Ralf Bartenschlager, Thomas Lengauer

**Affiliations:** 1 German Center for Infection Research (DZIF)–Saarbrücken Partner Site, Department of Computational Biology and Applied Algorithmics, Max Planck Institute for Informatics, 66123, Saarbrücken, Germany; 2 German Center for Infection Research (DZIF)–Cologne-Bonn Partner Site, Institute of Virology, University of Cologne, 50935, Cologne, Germany; 3 German Center for Infection Research (DZIF)–Heidelberg Partner Site, Department of Infectious Diseases, Molecular Virology, Heidelberg University, 69120, Heidelberg, Germany; 4 Institute for Virology, University Hospital Düsseldorf, Heinrich Heine University, 40225, Düsseldorf, Germany; 5 MVZ Medizinisches Infektiologiezentrum Berlin (MIB), 13353, Berlin, Germany; Centro de Biología Molecular Severo Ochoa (CSIC-UAM), SPAIN

## Abstract

The face of hepatitis C virus (HCV) therapy is changing dramatically. Direct-acting antiviral agents (DAAs) specifically targeting HCV proteins have been developed and entered clinical practice in 2011. However, despite high sustained viral response (SVR) rates of more than 90%, a fraction of patients do not eliminate the virus and in these cases treatment failure has been associated with the selection of drug resistance mutations (RAMs). RAMs may be prevalent prior to the start of treatment, or can be selected under therapy, and furthermore they can persist after cessation of treatment. Additionally, certain DAAs have been approved only for distinct HCV genotypes and may even have subtype specificity. Thus, sequence analysis before start of therapy is instrumental for managing DAA-based treatment strategies. We have created the interpretation system geno2pheno_[HCV]_ (g2p_[HCV]_) to analyse HCV sequence data with respect to viral subtype and to predict drug resistance. Extensive reviewing and weighting of literature related to HCV drug resistance was performed to create a comprehensive list of drug resistance rules for inhibitors of the HCV protease in non-structural protein 3 (NS3-protease: Boceprevir, Paritaprevir, Simeprevir, Asunaprevir, Grazoprevir and Telaprevir), the NS5A replicase factor (Daclatasvir, Ledipasvir, Elbasvir and Ombitasvir), and the NS5B RNA-dependent RNA polymerase (Dasabuvir and Sofosbuvir). Upon submission of up to eight sequences, g2p_[HCV]_ aligns the input sequences, identifies the genomic region(s), predicts the HCV geno- and subtypes, and generates for each DAA a drug resistance prediction report. g2p_[HCV]_ offers easy-to-use and fast subtype and resistance analysis of HCV sequences, is continuously updated and freely accessible under http://hcv.geno2pheno.org/index.php. The system was partially validated with respect to the NS3-protease inhibitors Boceprevir, Telaprevir and Simeprevir by using data generated with recombinant, phenotypic cell culture assays obtained from patients’ virus variants.

## 1. Introduction

Infection with hepatitis C virus (HCV) is a major health problem worldwide. It is estimated that 130 to 150 million individuals are chronically infected with this virus [1]. Epidemiological studies have shown that persistent infection with HCV leads to a significantly increased risk of developing severe liver diseases, most notably liver cirrhosis and hepatocellular carcinoma (HCC) [2]. The incidence of HCC in HCV infected individuals is 15 to 20 fold higher than in HCV-negative individuals and, as a consequence, more than 350,000 people die from hepatitis C-related liver diseases each year [3].

HCV is an enveloped RNA virus with a positive-sense single stranded genome and belongs to the family *Flaviviridae* [4]. HCV infections are highly dynamic processes that are maintained by rapid production of new virions and continuous cell-to-cell spread. Model-based approaches suggest a virion production rate of 10^12^ virions/day [5,6]. Moreover, genome amplification by the HCV NS5B RNA-dependent RNA polymerase (RdRp) is characterized by a high error rate (~ 10^−3^ errors per round of replication [7,8]), due to the lack of a proof-reading mechanism. These two properties result in the high genomic variability of HCV that is reflected in the existence of seven distinct genotypes (1 to 7) with a pairwise nucleotide divergence (percentage of non-homologous genomic sites) of at least 30% and at least 67 distinct subtypes (e.g. 1a, 1b,…) with a pair-wise nucleotide divergence of at least 20% [9,10].

The face of HCV therapy has changed dramatically since 2011. Novel direct-acting antiviral agents (DAAs), designed to inhibit distinct steps in the HCV replication cycle have been approved in the EU and the US. Currently, three classes of DAAs are available: inhibitors of the NS3 protease, the NS5A replicase factor and the NS5B RdRp. The amino-terminal domain of NS3 associates with NS4A to form the NS3-4A serine-type protease complex that catalyzes the cleavage of the HCV polyprotein. NS5A plays multiple roles in the HCV replication cycle such as induction of the membranous replication factory, acting as a cofactor for HCV RNA replication, and supporting the assembly of infectious virus particles. The RdRp in NS5B is responsible for viral RNA amplification. DAAs lay the foundation for all-oral, interferon-free treatment regimens [11,12]. However, the specific DAA eligibility, resistance prevalence and efficacy of treatment depend on the HCV geno- and subtypes [13–18]. Treatment failure with DAAs has been associated with the selection of resistance-associated variants (RAVs) that become majoritary during therapy either by de novo generation or a consequence of selection from variants present at baseline [19–21]. Indeed, resistance mutations for different DAAs are detected in therapy-naïve patients [16,21,22]. In addition, resistance mutations in NS3 were shown to persist for months after cessation of therapy and even for years in case of NS5A resistant variants [20,21,23–25], thus reducing the success rate with subsequent treatments and also increasing the risk to spread new infections with DAA-resistant HCV variants [11]. The number of treatment failures and drug resistant variants is expected to increase within the next years through selection pressure imposed by DAA-based therapy [11]. The characterization of these variants and their impact on first-line and re-treatment strategies remains a great challenge.

We have developed geno2pheno_[HCV]_ (g2p_[HCV]_), a web-service that supports the analysis of HCV sequence data with respect to geno- and subtypes and possible resistance against licensed DAAs. g2p_[HCV]_ is a new member of the geno2pheno family, a set of web-based interpretation tools for analyzing sequences of hepatitis B virus and human immunodeficiency virus [26–28]. The subtyping algorithm of g2p_[HCV]_ accounts for all geno- and subtypes recognized by the International Committee for Taxonomy of Viruses [10]. The drug resistance analysis is based on a comprehensive set of rules that were collected from clinical and *in vitro* studies and were reviewed and carefully weighted by an expert panel. g2p_[HCV]_ can be freely accessed via an easy-to-use web interface and affords the export of the analysis in PDF format to facilitate communication and storage of results. g2p_[HCV]_ may be used by researchers and may help physicians in developing personalized treatment schedules. To evaluate g2p_[HCV]_, we used a selected number of HCV variants from patients suffering from therapy failure and conducted phenotypic assays to monitor drug sensitivity and replication fitness.

## 2. Materials and Methods

### 2.1. Geno2pheno[HCV] prediction tool

#### 2.1.1. Reference sequence set

For subtyping, a reference alignment of 191 reference sequences for seven genotypes including 82 assigned subtypes and 35 unassigned subtypes was obtained in February 2015 from the International Committee for Taxonomy of Viruses [10]. From this reference alignment we extracted genomic regions relevant for drug resistance. These include: the protease domain of NS3 (up to amino acid position 181 of NS3), the amphipathic α-helix and the D1 domain of NS5A (up to amino acid position 213 of NS5A), and the complete NS5B region. For each region and subtype we defined one sequence to be the default reference sequence (e.g. H77 for 1a, HCV-J for 1b, etc.). The default reference is used for subtyping and for reporting genetic variants.

#### 2.1.2. Query Sequence Processing

g2p only processes nucleotide sequences. Mixtures of nucleotides at an individual position can be included if they are coded as indicated by the International Union of Pure and Applied Chemistry (IUPAC) [http://www.bioinformatics.org/sms/iupac.html]. A query sequence that is submitted as input to g2p is processed as follows: (1) the genomic region is identified, (2) the geno- and subtypes are identified, (3) the nucleotide sequence is translated into an amino acid sequence and a list of amino acid variants is extracted, (4) the amino acid variants are subjected to the rule set to perform the drug resistance analysis. We now describe these processing steps in more detail.

**2.1.2.1. Identification of the genomic region:** To identify all the genomic regions present in the query sequence, g2p aligns the query sequence against the multiple sequence alignments of the NS3, NS5A and NS5B regions. For each genetic region, the system computes the alignment length and alignment quality. Alignment length is the number of columns in the multiple sequence alignment that do not contain any gaps. If the alignment length is less than 100, the query sequence is found to be of poor quality and it is not analyzed any further. Otherwise, the sequence similarity between the aligned query sequence and each reference sequence is computed. Sequence similarity is defined as the number of aligned characters that match divided by the alignment length. If sequence similarity is less than 65% the query sequence most likely contains many sequencing errors and is not analyzed any further. To summarize, regions of the query sequence are identified that correspond to NS3, NS5A and NS5B, and then all the query regions that satisfy the quality checks are analyzed.

**2.1.2.2. Geno- and subtypes prediction:** it is carried out individually for each query region and is based on homology. The geno- and subtypes of the query region are determined by the geno- and subtypes of the reference sequence with which the query sequence has the highest sequence similarity (proportion of matching characters). For subtype 1a sequences, also the clade classification is provided [16,29]. Sequence similarities against all reference sequences are displayed in the results page. This method was validated with 177 sequences (see Materials and Methods, section Subtyping validation).

**2.1.2.3. The query region is translated into the corresponding amino acid sequence:** Depending on the settings (flag 3 selection) in the input page (use H77 or use the most similar reference sequence), all substitutions with respect to the corresponding reference are extracted. Nucleotide ambiguities of the query sequence are processed accordingly and might result in several possible amino acids present at a single position (denoted by, e.g. I170IV). Note that for the amino acid positions we always refer to the sequence H77 as a numbering reference (GenBank Accession number AF011751). At the end of this step, for each query region, the system generates a list of amino acid substitutions.

**2.1.2.4. Rule set:** An extensive literature survey was performed in order to obtain a comprehensive summary of the knowledge on drug resistance to NS3 inhibitors (Asunaprevir, Boceprevir, Grazoprevir, Paritaprevir, Simeprevir, and Telaprevir), NS5A inhibitors (Daclatasvir, Elbasvir, Ledipasvir, and Ombitasvir), and NS5B inhibitors (Dasabuvir and Sofosbuvir) [20,30–73]. The final rule set was selected by a panel of experts. Each rule is represented by a Boolean expression (see next paragraph) and is associated with a list of geno- and subtypes to which the rule applies, a summarizing drug resistance prediction (see Table 1), and references to the relevant literature and the levels of evidence. The levels of evidence were established similarly to the classification system used by the European HIV Drug Resistance Guidelines: I = based on at least one prospective randomized study using surrogate markers e.g. viral load; II = based on at least one retrospective study; III = expert opinion based on scientific evidence derived from other clinical and in vitro observations.

**Table 1:** Description of the summarizing drug resistance prediction.

Rank	Resistance prediction	Description
1	resistant	The provided sequence contains at least one of the so far known resistance-associated mutations for this drug class (see “Rules”-tab).
2	possibly resistant	The provided sequence contains at least one mutation related to resistance but whose specific fold change/therapy failure association has not been determined yet (see “Rules”-tab).
3	substitution on scored position	The provided sequence contains mutation(s) in (an) amino acid residue(s) related to resistance but whose specific fold change/therapy failure association has not been determined yet (see “Rules”-tab).
4	susceptible	The provided sequence contains none of the so far known resistance-associated mutations for this drug class (see “Rules”-tab).
5	not licensed for genotype	The drug is not licensed for the predicted genotype.

Each resistance rule is either a simple rule like pos_1_AA_1_ (e.g. 155K) or one of the following compound rules:

1. pos_1_AA_1_ or pos_1_AA_2_ or … or pos_1_AA_n_ (e.g. 168A or 168H or 168T or 168Y)
2. pos_1_AA_1_ and pos_2_AA_2_ and … and pos_n_AA_n_ (e.g. 28M and 31F)

where AA_i_ indicates the amino-acid observed in the query at position pos_i_.

#### 2.1.3. Applying resistance rules to the query sequence

For the major geno- and subtypes of HCV (1a, 1b, 2a, 2b, 3a, 4a and 4d), the procedure for determining drug resistance is as follows. Flag 4 in the input page allows for the selection of resistance rule set. The default option enables the specific resistance rules that are applicable for the geno- and subtypes and region of the query. If the option “ignore subgenotype for drug resistance prediction” is selected, then all resistance rules applicable to the region of the query will be used.

The application of each rule in the list to the amino acid substitutions present in the query results in one of the following cases. The cases are listed in increasing order of susceptibility to the drug corresponding to the rule.

1. The rule applies fully. All amino acid substitutions required by the rule are present in the query. The associated drug resistance prediction is either “possibly resistant” or “resistant”. This is determined by the corresponding entry in the rules table.
2. The rule applies partially. This occurs when the resistance rule is a complex rule of type 2 and only some of the variants are present in the query. The associated drug prediction is “rule applies partially”.
3. There is a substitution at a resistance conferring position but the observed substitution is not known to confer resistance. The associated drug prediction is “substitution on scored position”.

For rare geno- and subtypes there is not sufficient clinical or phenotypic-assay based evidence to confidently make resistance predictions for any observed substitution. However it is possible that a substitution in a rare geno- and subtype may be of clinical importance if it is known to confer resistance in a closely related common subtype. In order to report such substitutions in rare geno- and subtypes g2p first identifies the common geno- and subtypes that is most similar to the query by homology. For instance if the query has been geno- and subtyped as 4k, a rare geno- and subtypes, then the common geno- and subtypes will be 4d, the most similar common geno- and subtypes. Subsequently it is checked which rules fully apply for the common geno- and subtypes. The associated drug prediction is “resistance-associated mutation (RAM) in related common geno- and subtypes”.

#### 2.1.4. Validation of subtyping

1684 non-recombinant full genome sequences annotated with geno- and subtypes were downloaded from the Los Alamos HCV Sequence Database [74]. All sequences that are contained in the genotyping reference set were removed. Additionally, the sequences with the accession numbers AY878650, AY878651, KC197235, and KC197240 were also excluded (due to likely incorrect geno- and subtypes annotations). From the remaining set of sequences at most 20 sequences for each geno- and subtypes were randomly selected. This resulted in a test set of 177 full genome sequences covering the following 33 subtypes: 1a, 1b, 1c, 2a, 2b, 2c, 2i, 2j, 2k, 2m, 3a, 3b, 3i, 4a, 4d, 4f, 4l, 4m, 4n, 4o, 4r, 5a, 6a, 6b, 6e, 6f, 6i, 6l, 6m, 6n, 6o, 6t, and 6v.

We tested our homology-based subtyping approach for different lengths of the query sequence (50, 100, 200, 300, 500, 700, and 900 base-pairs) constructed at randomly selected genomic positions to see which length of the input sequence would allow for accurate subtyping. We further expanded the test set by introducing random nucleotide mutations to the sequences at different error rates: 0%, 10%, 20%, or 30% of the sequence positions. An error rate of x% means that nucleotides at x% of randomly selected positions were substituted with an arbitrary other nucleic acid.

### 2.2. Phenotypic resistance determination

For validation of the g2p predictions we used 11 samples from 11 patients included in the PEPSI Study The samples displayed different patterns of resistance-associated mutations (RAMs). Phenotypic resistance assays to the protease inhibitors boceprevir (BOC), telaprevir (TVR) and simeprevir (SMV) was conducted by using a method described elsewhere [75]. In brief, HCV RNA was isolated from the blood samples using the Magna Pure Systems (Roche) according to the manufacturer’s protocol. RT-PCR (One Step RT-PCR Kit, Qiagen, Hilden, Germany) was performed as previously described [76], but with HCV subtype-specific primers. NS3/protease amplicons were purified and inserted into the subgenomic HCV replicon pFKi341-PiLucNS3-3'_ET [77] that was modified to contain ClaI and AscI restriction sites [75]. These were used to insert NS3-specific amplicons obtained with patient sera. Insert sequences were checked by sequencing (GenBank Accession Numbers: KP409203-KP409213). Then, Replicon-encoding plasmid vectors were used for *in vitro* transcription and replicon RNAs were transfected into Huh7-Lunet cells by electroporation [78]. Different concentrations of the drugs were added to the transfected cells and replication was determined by luciferase assay [78]. All measurements were performed in triplicate. IC_50_ values were calculated using the GraphPad Prism software package by applying non-linear regression fit curves. The mean IC_50_ value was then normalized to the IC_50_ values of the corresponding reference construct and expressed as mean fold-change IC_50_ value.

#### 2.2.1. Ethics statement

All patients enrolled in the PEPSI Study gave their written consent allowing the use of their blood samples for scientific purposes. The PEPSI Study has been approved by the ethics committee of the Medical Council North Rhine (Ärztekammer Nordrhein, Germany), No. 2012048.

## 3. Results and Discussion

### 3.1. Geno2pheno_[HCV]_ web interface

**Fig 1:**
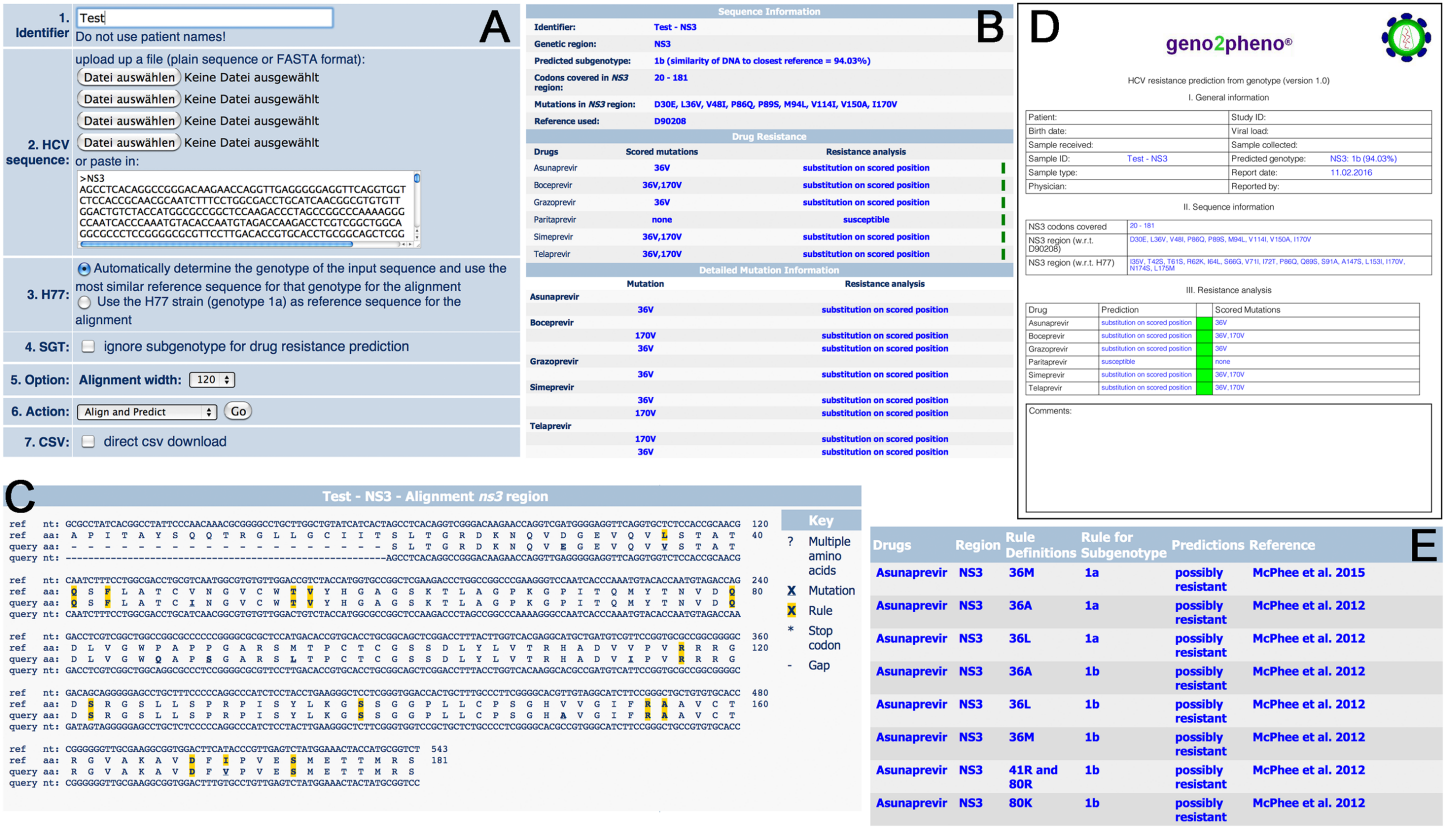
Multiple snapshots of the geno2pheno_[HCV]_ web interface. (A) The input page that allows the uploading of the sequence data and the configuration of the analysis. (B) The prediction sub-page that summarizes the subtype and drug resistance analysis. (C) The alignment sub-page (D) The drug resistance rule set as reference. (E) The PDF output to facilitate communication and storage of results.

The web-service geno2pheno_[HCV]_ (http://hcv.geno2pheno.org/index.php) was created to predict clinically relevant phenotypes based on viral sequence data. The web interface provides several pages, namely the input, results, rules, reference, contact and team pages (Fig 1).

#### 3.1.1. Input page

In the input page a user can enter (1) a sequence identifier that is displayed throughout the data analysis, (2) up to eight query sequences in FASTA format, (3) the H77 flag that specifies whether the list of amino acid substitutions should be listed with respect to H77 or with respect to the subtype specific reference, (4) the subtype flag which determines whether only the rules specific for the subtype inferred from the input sequence should be used or whether all rules should be used, (5) the alignment width used for the graphical representation of the alignment, (6) the CSV flag which allows the user to download the results as a CSV file, and (7) the action menu which allows the user to load a set of sample sequences or start the analysis.

#### 3.1.2. Results page

Upon pressing the action button “Align and Predict” in the input page, g2p_[HCV]_ performs the analysis and automatically switches to the result page. The result page offers one subpage for each query sequence with labels ranging from “1” up to “8”. Each of these so-called sequence pages further contains three subpages, the alignment subpage, the prediction subpage, and the subtype subpage.

The alignment sub-page provides visual representation of the nucleotide and amino acid sequence alignments of the NS3, NS5A, and NS5B regions of the query to the respective reference sequence. The alignment contains visual markers for RAMs to indicate mutations with respect to the selected reference sequence.

The prediction subpage contains the following three tables: (1) sequence information, (2) drug resistance prediction, and (3) detailed mutation information.

**3.1.2.1. The sequence information table:** it contains the sequence identifier (extracted from the FASTA header and combined with the identifier provided at the input page), the predicted subtype (in parentheses we provide the sequence similarity at the nucleotide level to the closest reference sequence) and the clade classification for subtype 1a sequences, the amino acid positions covered by the query, (in parentheses we indicate whether there are positions relevant to drug resistance that are not covered by the query), the list of amino acid substitutions with respect to the selected reference, and the GenBank accession number of the selected reference.

**3.1.2.2. The drug resistance table:** it contains the summary result of the drug resistance prediction. It has one row for each drug associated with the genomic region(s) identified in the query sequence. Each row contains the overall resistance prediction (see Table 1 for a detailed description) and the list of amino acid substitutions relevant for the prediction (so called scored mutations). The overall resistance prediction is shown in the right column as a colored square: green for “substitution on scored position” and “susceptible”, yellow for “possibly resistant” and red for “resistant”. The drug overall resistance prediction is the worst prediction among the resistance predictions corresponding to all scored mutations for that drug. For example if there are three scored mutations for a drug with the resistance predictions “resistant”, “possibly resistant” and “substitution on scored position” then the overall resistance prediction is “resistant”. If no scored mutations are found for a drug then the overall resistance prediction is “susceptible”. If the drug is not licensed for the subtype of the query then, instead of a resistance prediction, the message “drug not licensed for subtype” is displayed, and no color is provided for the overall resistance.

**3.1.2.3. The detailed mutation information table:** it contains one row for each scored mutation and lists its resistance prediction.

The genotype sub-page contains the list of sequence similarities of the query sequence with respect to all reference sequences. The list is sorted in the order of decreasing sequence similarity and may be helpful in assessing the reliability of the subtyping result. The genotype subpage also includes a “Download PDF” button to get a full report PDF that can be filled in for medical records.

#### 3.1.3. Rules page

The complete set of rules used for the drug resistance predictions is provided in the rules page. Each row corresponds to a rule associated with drug resistance. The entries of each row are (1) drug for which the rule is applicable, (2) target HCV protein, (3) the resistance rule provided as a Boolean expression, (4) the list of geno- and subtypes for which the rule is applicable, (5) the resistance prediction, (6) a list of scientific references from which this rule was derived and, (7) the evidence level qualifying the amount of clinical and phenotypic evidence that supports this rule.

#### 3.1.4. References, contact, and team page

The Reference page contains the full description of all references cited in the Rules page. The Contact page provides contact information regarding g2p_[HCV]_. Please do not hesitate to let us know if you find our service useful or if you run into any issues using our service. The Team page lists all the institutions and collaborators instrumental in the creation, maintenance and updating of g2p_[HCV]_.

### 3.2. Subtyping validation

A test set of 177 full genome HCV sequences of 33 different subtypes was compiled from the Los Alamos HCV sequence database. The detailed results of the subtyping validation are provided in S1 and S2 Figs. In short, we found that subtyping results are reliable if the sequence length is at least 300 base pairs (irrespective of genomic location). This resulted in 100% accuracy on our test set for the genomic regions encoding NS3 and NS5A independent of the error rate. Thus, even after flipping 30% of the bases the correct genotype could always be inferred. Subtyping accuracy for NS5B with a sequence length of at least 300 bases amounted to 97.1% to 98.3% depending on the error rate. We also analyzed the sequence similarity to the closest subtype which is provided as a quality criterion for the subtype predictions. For sequence lengths of at least 300 and error rates of at most 10%, 4202 of 4248 (98.9%) predictions exceeded a sequence similarity of 80%. Only 3 of the 4202 (0.07%) predictions that exceeded a sequence similarity of 80% were incorrect. The true subtypes for the incorrect predictions are 6n, 6o, and 6n; and the predicted subtypes are 6o, 6a, and 6e, respectively. The remaining 3999 (99.93%) predictions were correct. On the other hand, 46 of 4248 predictions had a sequence similarity that was less than 80%. Of these 22 (47%) predictions were correct and 24 (53%) were incorrect. Thus, homology-based subtyping of at least 300 base-pairs long sequences was found to be reliable on our test set for cases where the sequence similarity to the closest subtype exceeded 80%.

#### 3.2.1. Validation of predictions of drug resistance by using a phenotypic assay

**Fig 2:**
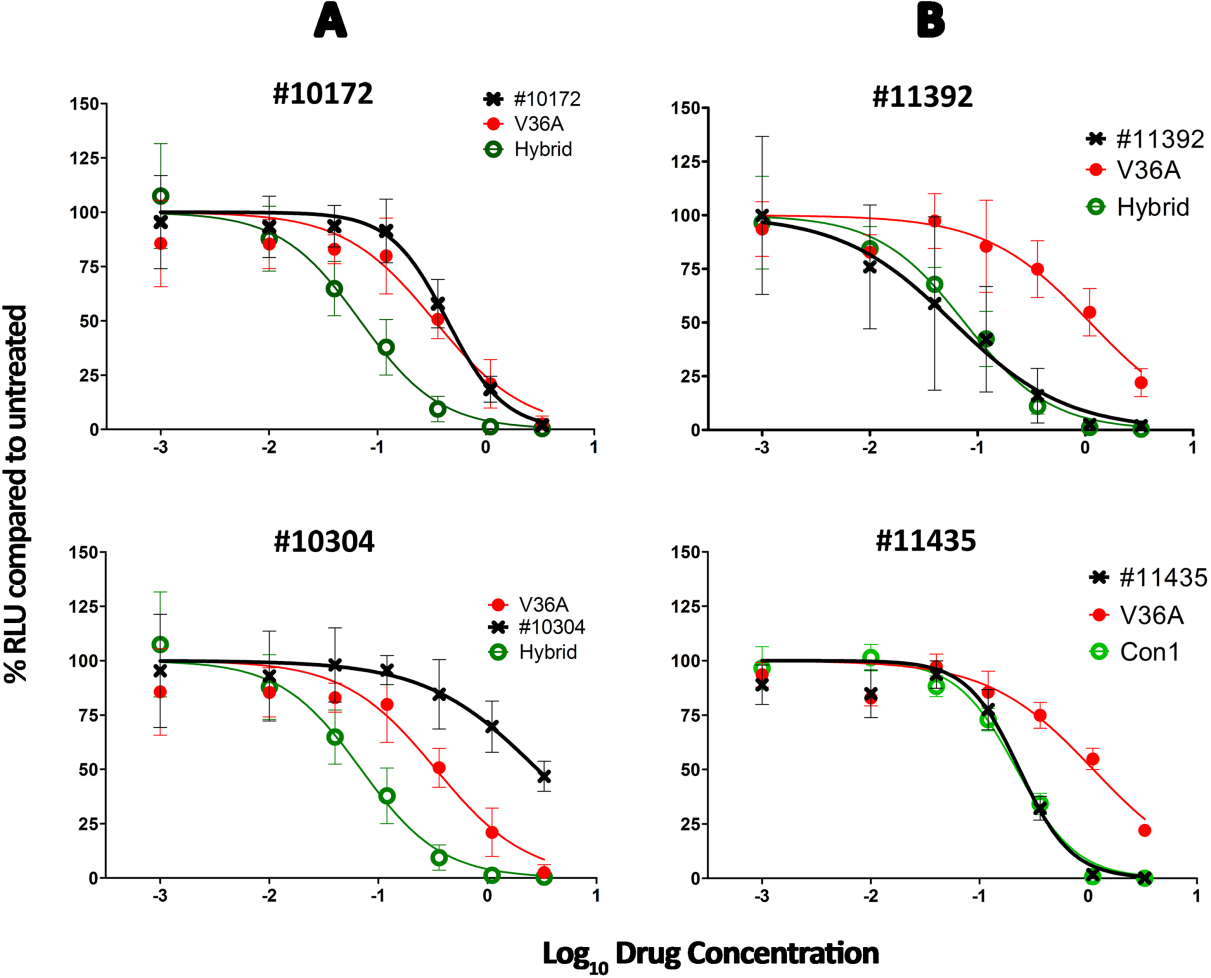
Examples of phenotypic measurements of PI susceptibility. The curves in green correspond to the susceptible controls (*Hybrid /Con1)*, those in red to the resistant construct *36A*, and the black ones to the specific sample. A) Samples #10172 and 10304 are resistant to BOC; B) Samples #10172 and 10304 are susceptible to TVR.

NS3-specific sequences were amplified from 11 patient sera and the amplicons were inserted into a subgenomic HCV replicon of the isolate Con1 engineered to allow easy transfer of NS3 amplicons via unique restriction sites. In this way, for each patient sample an NS3-specific library contained in a subgenomic replicon was generated and transfected into Huh7-Lunet cells. Their resistance to the NS3 protease inhibitors BOC, TVR and SMV was determined (Fig 2) and is expressed as “Fold-Change” (FC; Table 2). These results were compared to the predictions obtained with geno2pheno_[HCV]_. We found that phenotypic resistance determination with the replicon system correlated well with the corresponding genotypic resistance prediction by geno2pheno_[HCV]_. For clinical purposes it is important that samples detected to be highly resistant (FC ≥ 10) by phenotypic assays were also predicted as such by geno2pheno_[HCV]_.

**Table 2:** Phenotypic FC determinations.

Sample/Library	RAMs	BOC		TVR		SMV	
		g2p	FC assay	g2p	FC assay	g2p	FC assay
**Con1**	132V	S	1.0	S	1.0	S	1.0
**Hybrid**	-	S	1.0	S	1.0	S	1.3
**36A**	36A+132V	R	2.4	R	7.5	PR	1.0
**9712**	36A+132L+170V	R	1.5	PR	2.2	R	1.3
**10172**	36L	R	6.3	PR	6.3	PR	2.0
**10304**	36M+155K+170V	R	63.4	R	21.2	R	117.3
**10615**	80L	S	1.3	S	1.0	S	1.2
**11392**	122G+174S	S	0.3	PR	0.8	S	0.7
**11429**	132V	S	0.7	S	0.9	S	0.5
**11435**	41H+117H+132V	S	1.5	PR	1.0	S	0.4
**11610**	36M+80K+155K+174S	R	31.8	R	25.9	R	5.5x10^7^
**12292**	80K+132V+174S	S	1.8	PR	1.4	R	2.2
**12476**	174S	S	1.1	PR	2.8	S	2.0
**12516**	80K	S	1.6	S	0.5	R	9.1

g2p: S = susceptible; PR = possibly resistant; R = resistant; FC assay: fold change calculated from the phenotypic resistance assay

### 3.3. Use cases

The first version of the geno2pheno_[HCV]_ was made available for scientific use in March 2011 and has been regularly updated. The current g2p_[HCV]_ version (Oct 21^st^, 2015) is based on a rule set that incorporates state-of-the-art knowledge, is hand curated by the authors, and is regularly updated to account for novel developments. g2p_[HCV]_ can be useful in a variety of scenarios. In the following we describe two typical use cases.

#### 3.3.1. Case 1: patient 15170

A virus from a treatment-naïve patient was subtyped as 1a. Planned treatment for this patient was a combination therapy of Sofosbuvir plus Simeprevir. Resistance analysis performed with g2p_[HCV]_ revealed the presence of the Q80K mutation in the NS3 region. The Q80K mutation has been associated to lower response and also led to SMV resistance in phenotypic assays [79–82]. In addition, SMV fold changes of resistance up to 11 have been detected *in vitro* [37,38,50]. Due to this evidence another treatment strategy was chosen and the patient was subjected to a 12-week treatment with Sofosbuvir plus Ledipasvir, as the patient sequence did not show any resistance to these two drugs. The patient achieved sustained virological response.

#### 3.3.2. Case 2: patient 16083

A virus from a treatment naïve HCV was subtyped as 1a. The patient was then planned for a combination therapy of Paritaprevir, Ombitasvir and Dasabuvir. Resistance analysis followed by interpretation with g2p_[HCV]_ revealed the presence of the Q80K mutation in the NS3 region and 444D+556G in the NS5B region. The mutations 444D and 556G are described to confer resistance to Dasabuvir [64]. Consequently, the patient started treatment of Sofosbuvir plus Ledipasvir. 12 weeks after starting treatment (last value available), the viral load was still below the limit of detection.

### 3.4. Statistics of site usage

**Fig 3:**
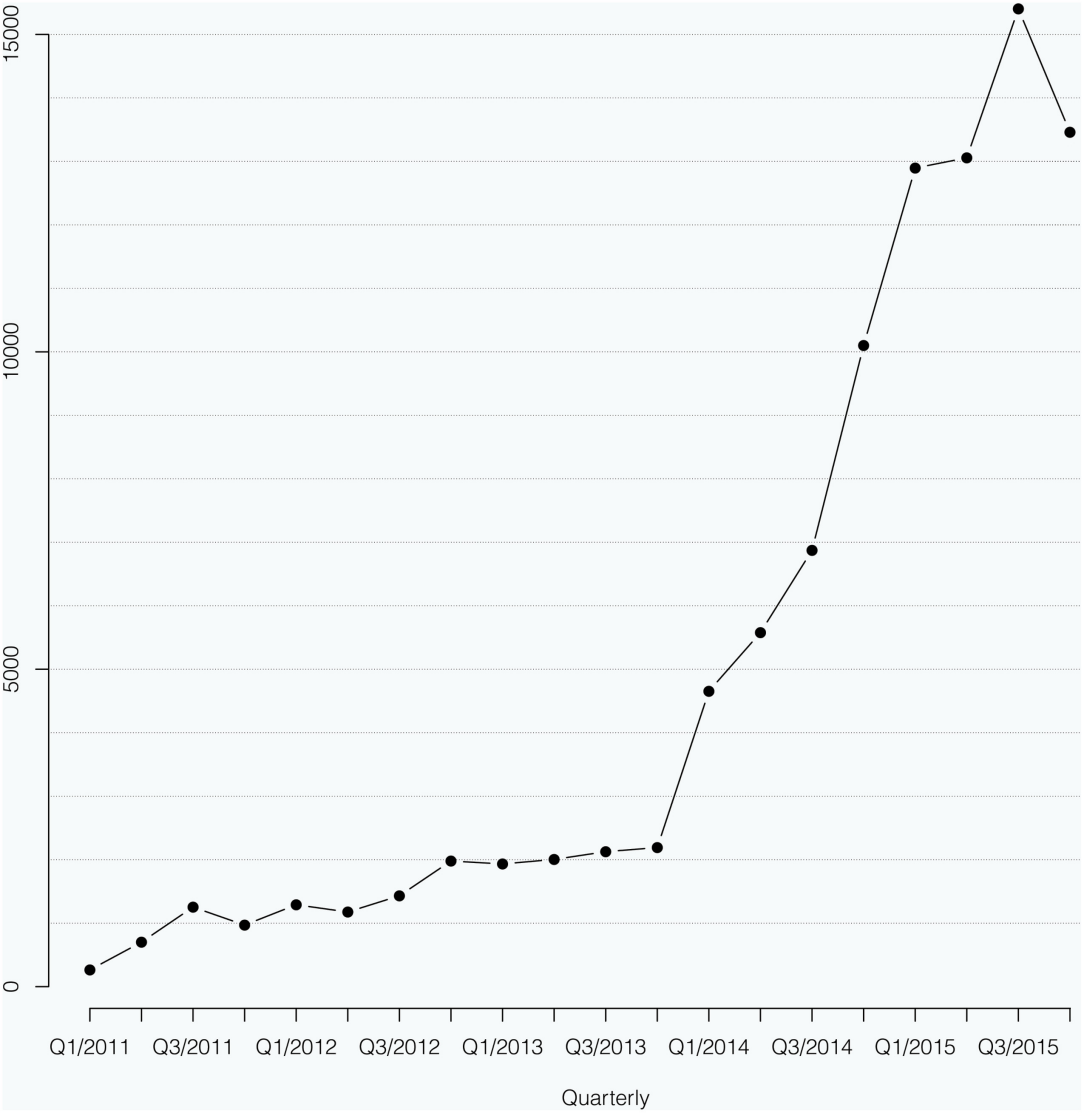
Cumulative number of queries per quarter that was submitted by users since the launch of the web-server g2p_[HCV]_.

We tracked the number of unique queries per day that were submitted to g2p_[HCV]_ since its launch in March 2011. We found that geno2pheno_[HCV]_ is a popular tool which received an average of 4600 queries per month in 2015. See Fig 3 for the cumulative queries per quarter from March 2011 up till December 2015.

### 3.5. Conclusion

To our knowledge, we present the first and only freely available web-service that provides an analysis of HCV sequence data with respect to subtype and simultaneously drug resistance. The service can interprete baseline drug resistance mutations and can be helpful in optimizing antiviral therapy.

We are committed to continuously updating g2p_[HCV]_ when novel drugs or resistance patterns are available. In addition, our access to phenotypic resistance determination assays will permit us to further validate the system but also to test mutations in target genes whose role in resistance is not clearly elucidated.

For the future, we also see high potential in the integration of additional host markers into g2p_[HCV]_ to further improve treatment recommendations. g2p_[HCV]_ can freely be accessed at http://hcv.geno2pheno.org/index.php.

## Supporting Information

S1 Fig Sequences subtyped against sequence length for each setting of genetic region and error rate. Each panel plots the number of sequences that were subtyped correctly (pink) and incorrectly (blue). For each genetic region, contiguous sequences of the specified length were randomly sampled and x% (error rate) of sequence characters were substituted with another nucleotide. The subtype for this sequence was given by subtype of the reference sequence that was most similar (%matches) to the sequence. Results are shown for 100 sequences constructed for each setting of error rate, sequence length and genetic region.
(TIF)

S2 Fig Similarities between the sequences and the reference of the correct subtype. Each panel shows the similarity between the query sequence and the reference of the correct subtype, against sequence length for each setting of genetic region and error rate. Cases where the sequence was subtyped correctly are shown in pink and the rest are shown in blue. For each genetic region, contiguous sequences of the specified length were randomly sampled and x% (error rate) of sequence characters were substituted with another nucleotide. The subtype for this sequence was given by subtype of the reference sequence that was most similar (%matches) to the sequence. Results are shown for 100 sequences constructed for each setting of error rate, sequence length and genetic region.
(TIF)
